# The longitudinal relationship between school climate and Internet gaming addiction among Chinese children: a moderated mediation model

**DOI:** 10.3389/fpsyt.2025.1662888

**Published:** 2025-09-09

**Authors:** Chang Wei, Qian Xu

**Affiliations:** ^1^ School of Arts and Sciences, Guangzhou Maritime University, Guangzhou, China; ^2^ College of Preschool Education, Guangzhou Preschool Teachers College, Guangzhou, China

**Keywords:** children, school climate, deviant peer affiliation, sensation seeking, internet gaming addiction

## Abstract

**Introduction:**

School climate is associated with children’s Internet gaming addiction; however, the mechanisms underlying this association have been largely unexplored. Based on the ecological systems theory, the social learning theory, and the diathesis-stress model, we examined the association between school climate and children’s Internet gaming addiction, as well as the mediating role of deviant peer affiliation and the moderating role of sensation seeking in this relationship.

**Methods:**

The study involved 419 Chinese children participating in a two-wave longitudinal investigation conducted over the course of one year. Among the participants, 54.4% were boys and 45.6% were girls. The mean age of the participants at Wave 1 was 10.70 years (SD = 0.58). We used SPSS 27.0 to generate descriptive statistics and correlations. We adopted Model 4 of the PROCESS for SPSS to examine the mediation model. We used Model 14 of the PROCESS for SPSS to examine the moderated mediation models.

**Results:**

The results showed that school climate was negatively correlated with Internet gaming addiction among children one year later. Deviant peer affiliation mediated the pathway from school climate to Internet gaming addiction, and this mediating pathway was moderated by sensation seeking.

**Conclusion:**

These findings demonstrated the individual differences in the association between school climate and Internet gaming addiction, which has implications for the prevention of Internet gaming addiction among children.

## Introduction

According to the Fifth National Survey Report on Internet Use among Minors (1), there were 193 million underage Internet users in China in 2022, and the proportion of internet games reached 67.8%. The internet plays a significant role in the lives of children and adolescents. They not only use it for entertainment and to pass the time but also to explore their identities. Moreover, the internet provides them with opportunities to stay connected with peers, make new friends, and fulfill their need to belong to a peer group (2). Internet gaming addiction refers to the persistent and recurrent use of the Internet to engage in games, often with other players, resulting in clinically significant impairment or distress (3). Moreover, the DSM-5 lists nine diagnostic criteria for Internet gaming addiction: preoccupation with gaming, withdrawal symptoms, increased tolerance, unsuccessful attempts to control gaming, loss of interest in other activities, continued gaming despite problems, deception of others regarding gaming, using gaming to escape negative moods, and lose relationships/opportunities. Based on these criteria, to be diagnosed with Internet gaming addiction, an individual must meet at least five of the nine criteria within the past 12 months. Internet gaming addiction is not only associated with mental health issues such as anxiety and depression in children and adolescents (4), but also related to self-harm and suicidal behaviors (5). Nevertheless, considering the ubiquity of Internet use in children’s daily lives, this study does not pathologize Internet use. Instead, it focuses on exploring the potential link between school climate and the tendency toward Internet gaming addiction.

### School climate and Internet gaming addiction

Previous studies have shown that a positive school climate is an important protective factor for the mental health development of children and adolescents (6, 7). School climate refers to the relatively long-lasting and stable environmental characteristics of a school that are experienced by members and have an important impact on members’ behavior (8). The ecological systems theory (9) proposes that schools are important microsystems influencing individual development. According to this theory, school climate as a protective factor (6, 7) may reduce the risk of Internet gaming addiction among children. Empirical research further supports this perspective, indicating that school climate can effectively reduce the risk of Internet gaming addiction among children and adolescents. (10, 11). For example, Chen (10) found in a longitudinal study of 1023 children that school climate significantly negatively predicted Internet gaming addiction six months later.

### Deviant peer affiliation as a potential mediator

The first half of the mediation pathway we examined was the impact of school climate on children’s deviant peer affiliation. According to the ecological systems theory (9), the school climate, as an important microsystem, can directly influence children’s behaviors. Previous research has shown that a positive school climate helps to suppress adolescents’ deviant peer affiliation, while a negative school climate may exacerbate adolescents’ deviant peer affiliation (12, 13). In a study of 1084 Chinese adolescents, Shi (13) found that school climate was significantly negatively correlated with deviant peer affiliation.

The second half of the mediation pathway we examined was the impact of deviant peer affiliation on children’ Internet gaming addiction. According to the social learning theory (14), children may learn to engage in Internet gaming by observing their friends’ behavior. In addition, empirical studies have also supported this view (11, 15). For example, Tian et al. (15) found that deviant peer affiliation was positively associated with adolescents’ Internet gaming addiction.

### Sensation seeking as a moderator

Sensation seeking is a personality trait characterized by the tendency to seek out varied, novel, complex, and intense sensations and experiences, and to be willing to take risks for the sake of such experiences (16, 17). Empirical research has demonstrated that sensation seeking is an important risk factor for Internet gaming addiction (15, 18, 19). For example, Hamid et al (18) found that sensation seeking was positively associated with Internet gaming addiction in a sample of 260 adolescents. Similarly, in a sample of 375 Chinese adolescents, Hu et al. (19) found that sensation seeking was significantly positively associated with Internet gaming addiction.

According to the diathesis-stress model (20), children with personality vulnerabilities (e.g., high sensation seeking) may develop Internet gaming addiction when exposed to deviant peer affiliation. Consistent with this view, empirical studies have documented that sensation seeking plays a moderating role in amplifying the risk of adverse environments for problem behaviors (21–24). For example, in a longitudinal study, Chen et al. (21) found that high sensation seeking amplified the effect of childhood emotional neglect on problematic mobile phone use in a sample of 1987 adolescents. Similarly, Rioux et al. (23) found that sensation seeking interacted with low parental knowledge to predict substance use in a sample of 230 adolescents.

### The present study

**Figure 1:**
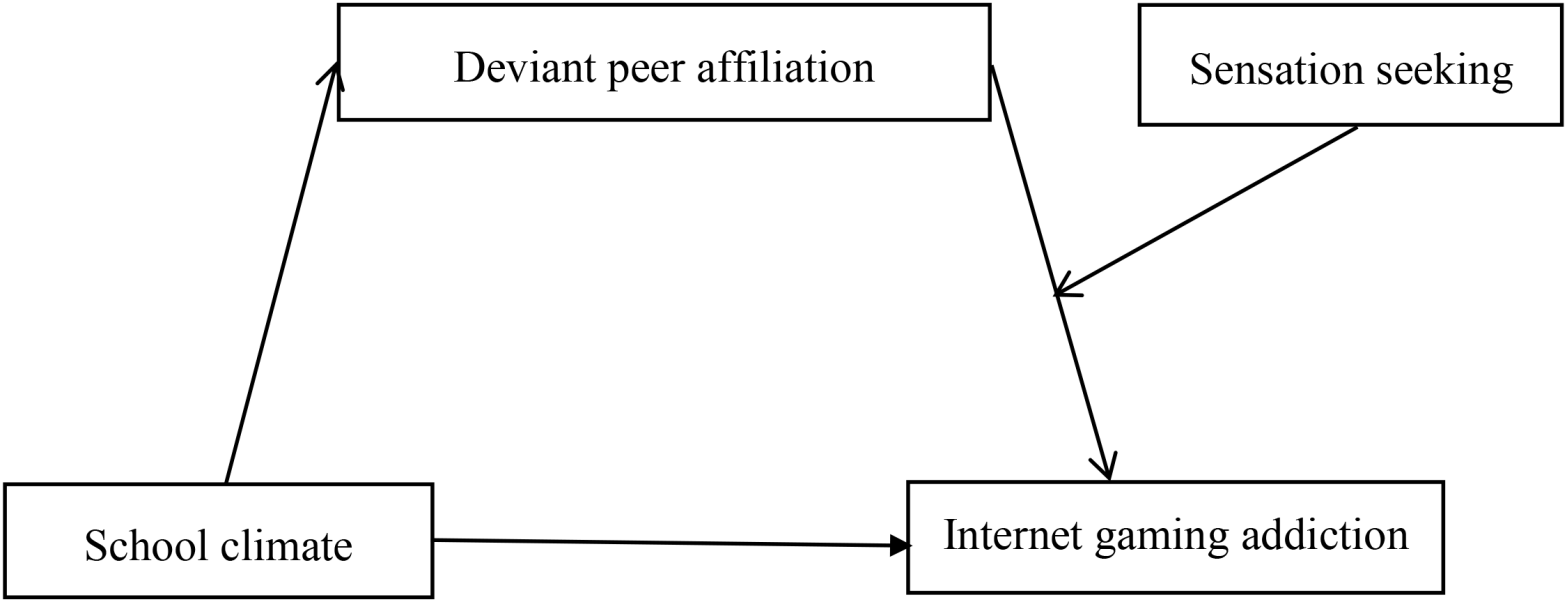
The proposed moderated mediation model.

Based on the ecological systems theory (9), the social learning theory (14), and the diathesis-stress model (20), we examined the association between school climate and children’s Internet gaming addiction, as well as the mediating role of deviant peer affiliation and the moderating role of sensation seeking in this relationship. Based on scientific literature and previous research findings, we hypothesized that there is a negative correlation between school climate and children’s Internet gaming addiction (Hypothesis 1). We further hypothesized that the association between school climate and children’s Internet gaming addiction may be mediated by deviant peer affiliation (Hypothesis 2), and that sensation seeking may moderate this mediating process (Hypothesis 3). **Figure 1** presents the proposed model.

## Method

### Participants

A total of 419 Chinese children participated in this two-wave longitudinal study, which was conducted with a one-year interval between waves. The participants were from fifth-grade primary school students, among whom 228 were boys (54.4%) and 191 were girls (45.6%). Additionally, 302 participants were from rural areas (72.1%), 117 were from urban areas (27.9%), 38 were only children (9.3%), and 381 were not only children (90.7%). The mean age of the participants in Wave 1 was 10.70 years (SD = 0.58), with an age range of 10 to 12 years.

### Measures

#### School climate

At Wave 1, school climate was measured using the Perceived School Climate Scale (25). This scale includes 25 items (e.g., “Students trust each other”) rated on a 4-point scale (1 = never to 4 = always). Mean scores were used for analysis, with higher scores indicating higher perceived school climate. In this study, Cronbach’s alpha was .89 at Wave 1.

#### Deviant peer affiliation

At both Wave 1 and Wave 2, deviant peer affiliation was measured with the Deviant Peer Affiliation Scale (26). This scale includes of 10 items to describe their friends (such as “internet addiction”). Each item is rated on a 5-point scale (1 = none to 5 = six or more). Mean scores were used for analysis, with higher scores indicating a higher level of deviant peer affiliation. In this study, Cronbach’s alpha was .82 and .77 at Waves 1 and 2, respectively.

#### Internet gaming addiction

At both Wave 1 and Wave 2, Internet gaming addiction was measured with the Internet Gaming Disorder Questionnaire (27). This questionnaire includes 9 items (such as “Do you feel the need to increase the amount of time spent gaming, play more exciting games, or use more powerful equipment in order to achieve the same level of excitement as before?”). Each item is rated on a 5-point scale (1 = never to 5 = very often). Mean scores were used for analysis, with higher scores indicating a higher level of Internet gaming addiction. In this study, Cronbach’s alpha was .84 and .84 at Waves 1 and 2, respectively.

#### Sensation seeking

At Wave 2, sensation seeking was measured using a six-item subset (28) from the Sensation Seeking Scale (29). This scale includes 6 items (e.g., “I enjoy novel and exciting experiences, even if they are a bit scary.”). Mean scores were used for analysis, with higher scores indicating higher levels of sensation seeking. In this study, Cronbach’s alpha was .83 at Wave 2.

#### Control variables

Children and adolescents show significant gender differences in Internet gaming addiction, with boys being at a higher risk than girls (30). Additionally, Internet gaming addiction is associated with age (30, 31). Consequently, we controlled for age and gender in our analyses.

### Procedure

This study was approved by the Academic Ethics Review Board of school of Arts and Sciences, Guangzhou Maritime University. The inclusion criteria included having a written informed consent from the parents and the child’s agreement to take part. Children who refused to participate in the survey or were absent from school due to leave were excluded. Participants were assured that they had the option to leave the study at any time without facing any penalties, and that their responses would be kept confidential. The children filled out the survey in their classroom. To express gratitude, each participant was given a signature pen.

### Statistical analyses

We used SPSS 27.0 to generate descriptive statistics and correlations. We adopted Model 4 of the PROCESS for SPSS to examine whether deviant peer affiliation mediated the association between school climate and Internet gaming addiction. We used Model 14 of PROCESS for SPSS to examine whether sensation seeking moderated the association between deviant peer affiliation and Internet gaming addiction. Moreover, the present study incorporated gender and age as covariates in the analysis.

## Results

### Preliminary analyses

**Table 1** shows the means, standard deviations, and correlation coefficients for all study variables. School climate at Wave 1 was negatively associated with Internet gaming addiction at Wave 2. School climate at Wave 1 was negatively associated with deviant peer affiliation at Wave 2. Deviant peer affiliation at Wave 2 were positively associated with Internet gaming addiction at Wave 2. In addition, sensation seeking at Wave 2 were positively associated with Internet gaming addiction at Wave 2.

**Table 1:** Descriptive statistics and correlations for all variables.

Variable	1	2	3	4	5	6	7	8
1. Gender	1.00							
2. Age	0.14**	1.00						
3. SC at Wave 1	−0.04	−0.06	1.00					
4. DPA at Wave 1	0.15**	0.06	−0.21***	1.00				
5. DPA at Wave 2	0.03	−0.02	−0.24***	0.38***	1.00			
6. IGA at Wave 1	0.17***	0.04	−0.29***	0.30**	0.28***	1.00		
7. IGA at Wave 2	0.10*	−0.04	−0.29***	0.28***	0.40***	0.47***	1.00	
8. SS at Wave 2	−0.03	−0.12*	0.03	0.13**	0.14**	0.19***	0.22***	1.00
*Mean*	0.54	10.70	2.73	1.26	1.20	1.31	1.22	2.48
*SD*	0.50	0.59	0.53	0.42	0.35	0.41	0.38	1.23

Gender was dummy coded as 1 = male, 0 = female. SC, school climate; DPA, deviant peer affiliation; IGA, Internet gaming addiction; SS, sensation seeking. *p <.05. **p <.01. ***p <.001.

### Mediation effect of deviant peer affiliation

**Figure 2:**
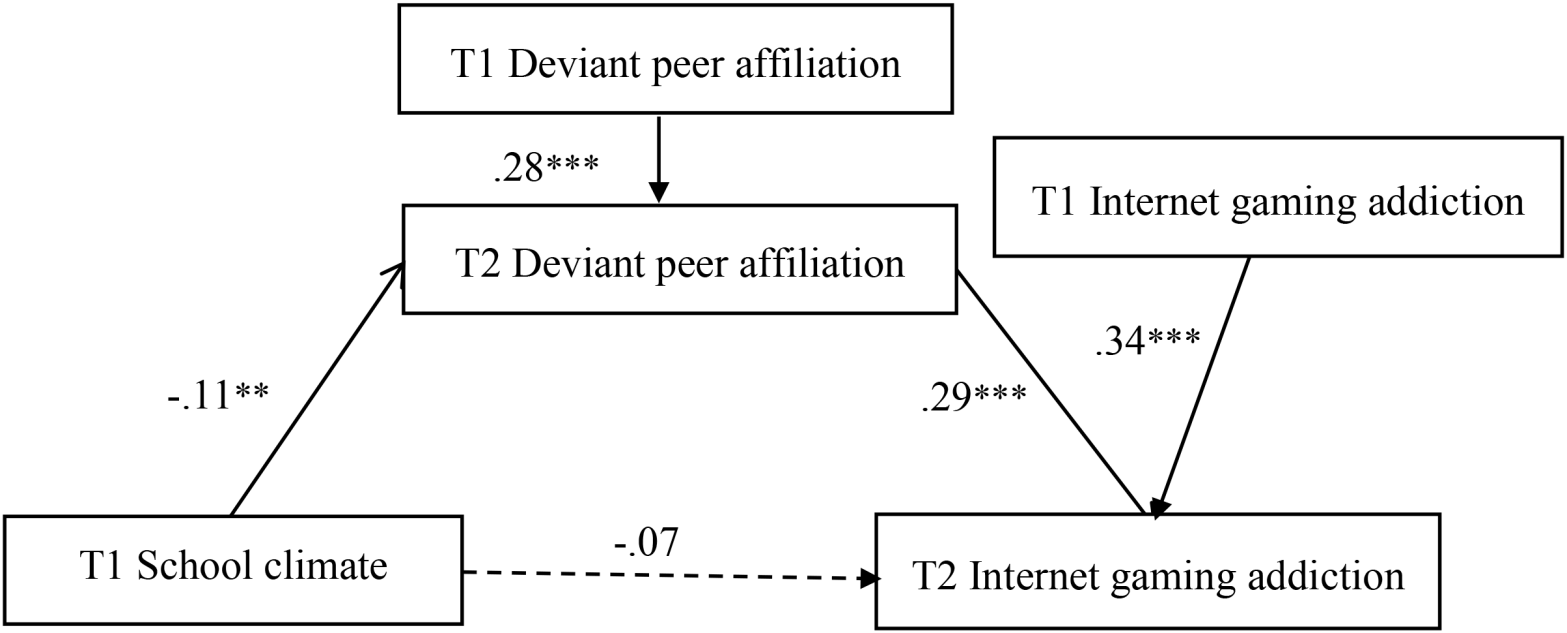
Model of the mediating role of deviant peer affiliation in the association between school climate and internet gaming addiction. The mediational analysis of deviant peer affiliation at Wave 2 in the association between school climate at Wave 1 and Internet gaming addiction at Wave 2. The dashed line indicates that the relationship is not significant. **p < .01. ***p < .001.

**Figure 2** shows the results obtained from the mediation model. After controlling for gender, age, deviant peer affiliation, and Internet gaming addiction at Wave 1, school climate at Wave 1 negatively predicted deviant peer affiliation at Wave 2 (*b* = −.11, *SE* = .04, *p* <.01), which in turn positively predicted Internet gaming addiction at Wave 2 (*b* = .29, *SE* = .06, *p* <.001). The bias-corrected percentile bootstrap method showed a significant mediating effect of deviant peer affiliation at Wave 2 in the relationship between school climate at Wave 1 and Internet gaming addiction at Wave 2 (indirect effect = −.033, *SE* = .014, 95% CI = [−0.063, −0.009]).

### Moderated mediation

**Figure 3:**
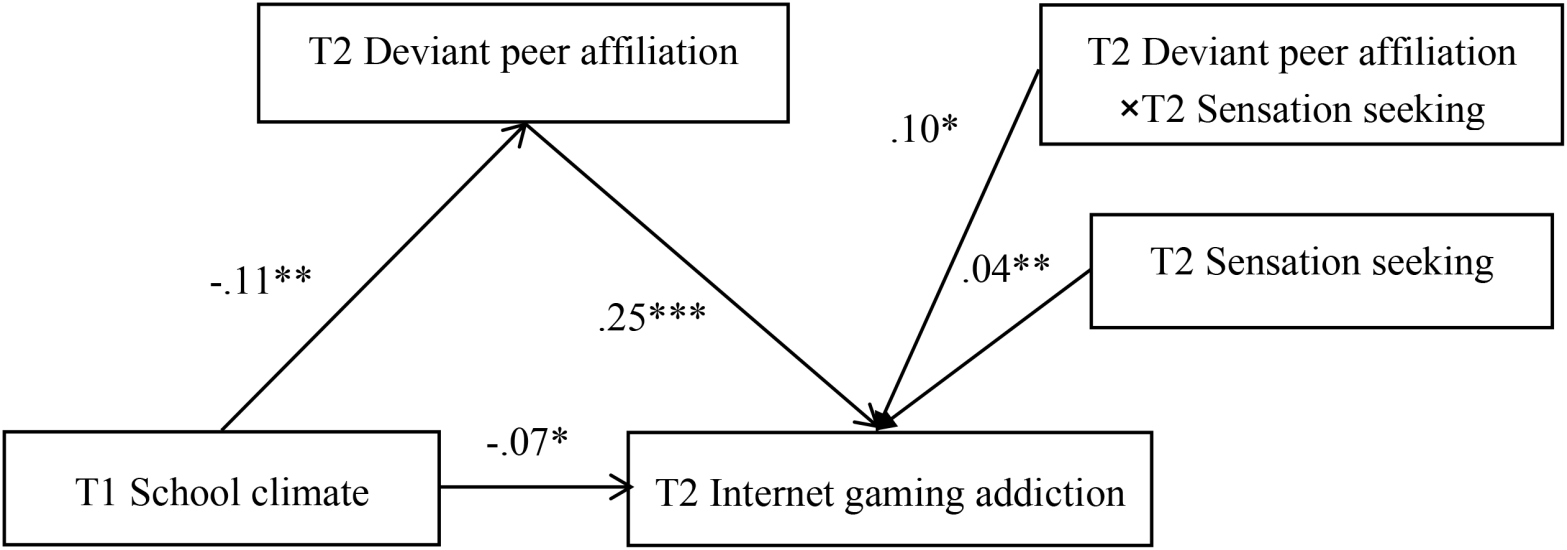
Model of the moderating role of sensation seeking on the relationship between deviant peer affiliation and internet gaming addiction. Image 3 model of the moderating role of sensation seeking at Wave 2 on the relationship between deviant peer affiliation at Wave 2 and Internet gaming addiction at Wave 2. The dashed line indicates that the relationship is not significant. *p < .05. **p < .01. ***p < .001.

**Figure 4:**
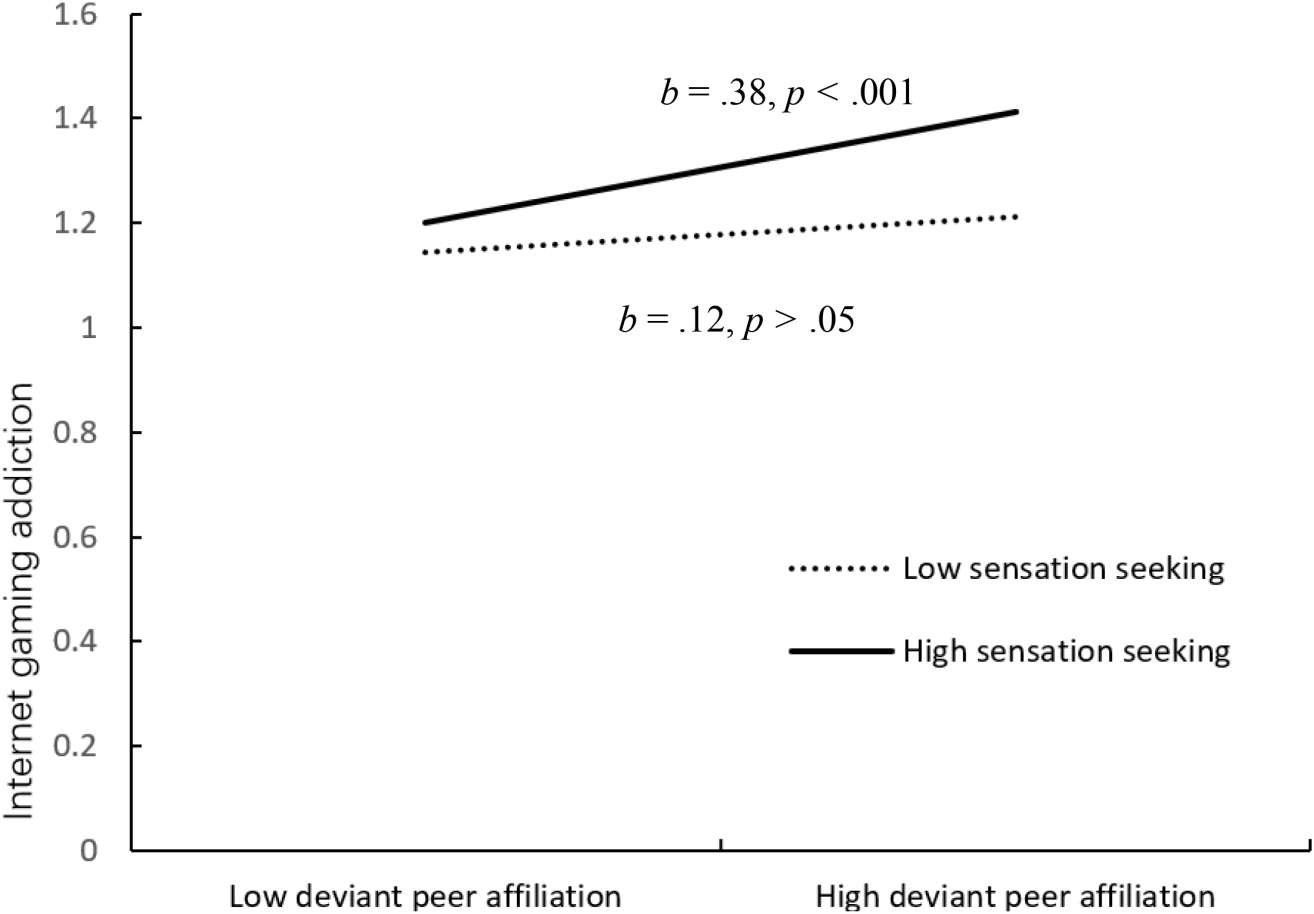
Interactive effect of deviant peer affiliation and sensation seeking on internet gaming addiction. *Note.* Sensation seeking as a moderator of the relationship between deviant peer affiliation and Internet gaming addiction.

**Figure 3** shows the results of the moderated mediation model. The results showed that sensation seeking at Wave 2 moderated the association between deviant peer affiliation at Wave 2 and Internet gaming addiction at Wave 2 (*b* =.10, *SE* = .04, *p* <.05). We conducted simple slope tests to better understand the results using sensation seeking as a moderator. As shown in **Figure 4**, when participants reported high sensation seeking, the association between deviant peer affiliation at and Internet gaming addiction was significant (*b* = .38, *SE* = .07, *p* <.001). However, for participants who reported low sensation seeking, this association was not significant (*b* = .12, *SE* = .09, *p* >.05). Therefore, the mediating effect of deviant peer affiliation on the association between school climate and Internet gaming addiction was significant in children with high sensation seeking.

## Discussion

Based on the ecological systems theory (9), the social learning theory (14), and the diathesis-stress model (20), we examined the association between school climate and children’s Internet gaming addiction, as well as the mediating role of deviant peer affiliation and the moderating role of sensation seeking in this relationship. This study included gender and age as control variables, both of which are known to significantly influence Internet addiction in children. By controlling these variables in the analysis, we were able to more clearly reveal the relationships among the primary study variables. As expected, we found that school climate was negatively correlated with children’s Internet gaming addiction. Moreover, deviant peer affiliation mediated the association between school climate and Internet gaming addiction, and sensation seeking moderated this indirect effect.

### Relationship between school climate and Internet gaming addiction

Consistent with hypothesis 1, this study found that school climate was significantly negatively correlated with Internet gaming addiction. That is, a positive school climate reduces the risk of children’s Internet gaming addiction. This finding is in line with previous research results, confirming that the school climate is an important protective factor against Internet gaming addiction in children and adolescents (10, 11). A positive teacher–student relationship, mutually supportive peer relationships, and an atmosphere that encourages student autonomy within the school environment can lead children to form a strong sense of attachment to their school. This sense of attachment is an important factor in curbing their Internet gaming addiction (7). Moreover, previous studies have shown that the school atmosphere can influence children’ sense of belonging at school (32). Belongingness is a fundamental psychological need for children. When they encounter setbacks in reality, they may turn to the online world for compensation. According to the perspective of compensatory internet use (33), children who feel a lack of belonging in real life may use the internet to fulfill their unmet needs. Previous studies have shown that adolescents with low school belongingness and high social media belongingness are more likely to rely on social media, thereby increasing their risk of social media addiction (34). In summary, this empirical study reveals a significant negative correlation between school climate and children’s Internet gaming addiction, further confirming the importance of school climate as a protective factor. The findings align with the ecological systems theory (9), reinforcing the significant impact of school climate as an ecological factor on the development of children.

### The mediating role of deviant peer affiliation

Consistent with hypothesis 2, the study found that deviant peer affiliation mediated the association between school climate and Internet gaming addiction. Specifically, school climate reduces the risk of Internet gaming addiction among children by reducing their deviant peer affiliation. Previous research has shown that deviant peer affiliation is an important mediating mechanism that links school climate to adolescent problem behaviors (11, 12). A positive school climate fosters strong teacher–student and peer relationships, which can reduce children’s involvement with deviant peers (13, 35). When children interact with deviant peers, they may learn excessive Internet gaming behaviors through observation, potentially leading to addiction. This empirical study elucidates how school climate impacts children’s Internet gaming addiction through deviant peer affiliation, offering a new perspective on understanding this phenomenon. Additionally, the study not only confirms the mediating role of deviant peer affiliation but also provides further empirical support for social learning theory (14), contributing research materials to the development of related theories. These findings not only enrich the existing research literature but also offer concrete intervention directions for schools and educators, namely, to reduce children’s deviant peer affiliation and thereby lower the risk of Internet gaming addiction by optimizing the school climate.

### The moderating role of sensation seeking

Consistent with Hypothesis 3, sensation seeking intensified the relationship between deviant peer affiliation and Internet gaming addiction, thereby further amplifying the indirect link between school climate and Internet gaming addiction. Specifically, deviant peer affiliation significantly positively predicts Internet gaming addiction, but only in children with higher levels of sensation seeking. This indicates that higher sensation seeking, as a personality vulnerability factor, amplifies the negative impact of deviant peer affiliation on children’s Internet gaming addiction. This empirical study reveals how sensation seeking moderates the relationship between deviant peer affiliation and Internet gaming addiction, thereby providing a deeper understanding of the pathways through which school climate influences Internet gaming addiction in children. This finding not only extends existing research but also offers a new perspective on understanding the complex mechanisms underlying Internet gaming addiction. Consistent with the diathesis-stress model (20), our results further confirm that sensation seeking can strengthen the indirect mechanism through which school climate affects children’s Internet gaming addiction via deviant peer affiliation. This discovery provides new empirical support for the development of related theories. Moreover, the results of this study are consistent with previous research, which has indicated that higher sensation seeking, as a risk factor, can strengthen the link between adverse environments and problem behaviors in children and adolescents (21, 24).

### Limitations and future directions

This study had several limitations. Firstly, the child sample in this study was primarily drawn from Hubei Province, China. Given the geographical limitation, this sample cannot fully reflect the overall characteristics of the entire child population in China. Therefore, future research may consider recruiting a larger and more representative sample across the country to further explore the generalizability of the study findings. Second, the data in this study were obtained from children’s self-reports, which are intrinsically subjective. As a result, the study findings may be affected by factors such as social desirability bias or inaccurate memory. To reduce these limitations, future studies may consider adopting diversified data-collection methods, such as integrating reports from parents, friends and other sources of information, to obtain more objective data. Third, this study tested deviant peer affiliation as the mechanism of association between school climate and Internet gaming addiction. Future research could consider other latent variables, such as school engagement (21) and school belonging (32). Fourth, we examined the moderating role of sensation seeking. Future research could consider other individual variables, such as impulsivity (19). Fifth, this study collected data at two time points over a one-year period. Future research could consider collecting data at multiple time points to uncover the long-term impacts among the variables.

### Implications for practice

This study has several important implications for prevention and intervention strategies. First, given that a positive school climate is an important protective for children’s Internet gaming addiction, it is possible to reduce children’s Internet gaming addiction by creating a good school climate, such as promoting communication between teachers and students, between classmates, and providing students with opportunities for autonomy (25). Second, given that deviant peer affiliation is the mechanisms that link school climate with Internet gaming addiction, it is possible to reduce children’s deviant peer interaction by actively carrying out interpersonal education activities (13), thereby reducing the risk of Internet gaming addiction. Third, considering that higher sensation seeking intensifies the indirect link between school climate and Internet gaming addiction, implementing emotion regulation strategies could help reduce sensation seeking (36), thereby lowering the risk of Internet gaming addiction.

## Conclusions

The results showed that school climate was negatively correlated with Internet gaming addiction among children one year later. Deviant peer affiliation mediated the pathway from school climate to Internet gaming addiction, and this mediating pathway was moderated by sensation seeking. These findings demonstrated the individual differences in the association between school climate and Internet gaming addiction, which has implications for the prevention of Internet gaming addiction among children.

## Data Availability

The original contributions presented in the study are included in the article/supplementary material. Further inquiries can be directed to the corresponding author.
